# Gender Differences in Affective and Evaluative Responses to Experimentally Induced Body Checking of Positively and Negatively Valenced Body Parts

**DOI:** 10.3389/fpsyg.2019.01058

**Published:** 2019-05-14

**Authors:** Julia A. Tanck, Silja Vocks, Bettina Riesselmann, Manuel Waldorf

**Affiliations:** Department of Clinical Psychology and Psychotherapy, Institute of Psychology, Osnabrück University, Osnabrück, Germany

**Keywords:** body checking, men, women, gender difference, valence

## Abstract

Body checking (BC) is defined as behavior aimed at gaining information on body shape, size, or weight. Besides its occurrence as a transdiagnostic symptom in eating disorders (EDs), BC is widespread in non-clinical populations. It is associated with body dissatisfaction and the development of body image disturbances and ED. Males and females differ in terms of body dissatisfaction and associated BC strategies. However, the question of a gender-related intensity of negative affect and state body satisfaction as reactions to BC remains open. Therefore, the present experimental study aimed to examine gender differences in affective and evaluative responses to BC. We tested *n* = 60 women and *n* = 60 men in a crossover design, implementing two experimental conditions and one control condition. In the negative body checking condition (NBC), participants received standardized instructions to check negatively valenced body parts. In the positive body checking condition (PBC), by contrast, participants were asked to check positively valenced body parts. The control condition (CBC) consisted of playing a computer game. Before and after checking of one’s own body, participants rated negative body-related affect, i.e., guilt, shame, and disgust, and state body satisfaction. The results indicate that both NBC and PBC led to an increase in negative affect for men and women. Eating pathology predicted negative affect after checking in NBC for both genders. Men reported a significantly higher state body satisfaction in all conditions, whereas only women showed decreased body satisfaction in NBC. These findings suggest that BC of any body part (i.e., positively or negatively valenced) leads to increased negative affect for both genders. Eating pathology significantly influences the affective responses to BC for both genders. Changes in state body satisfaction, however, only occur in females. An explanation might be that men generally report higher body satisfaction, resulting in a more stable body image. Females and males with pathological eating behavior seem to be more vulnerable to changes in negative affect. As negative affect is equally increased after NBC and PBC for both genders, BC might play a central role in the maintenance of body image disturbances in males and females.

## Introduction

Body checking (BC) behavior is known as the behavioral manifestation of an overconcern with shape, weight, and body size (Fairburn et al., 2003; Shafran et al., 2004). Episodes of BC normally last for only a brief period of time and are focused on disliked body parts (Shafran et al., 2004). Examples of such behaviors include feeling for bones, pinching flesh, measuring the size of multiple body parts, compulsive weighing, and social scanning (Williamson, 1990; Fairburn et al., 1999; Reas et al., 2002; Williamson et al., 2004). BC is associated with the development of a negative body image (Rosen et al., 1991; Rosen, 1997; Reas et al., 2002), which is observable across different body sizes as well as cultures (Dohnt and Tiggemann, 2006) and genders (Walker et al., 2012). A negative body image is known to be a key factor in the development and maintenance of clinical eating disorders (EDs; e.g., Stice and Shaw, 2002; Johnson and Wardle, 2005; Keel et al., 2005; Hildebrandt et al., 2010; Vartanian and Grisham, 2012), as individuals with a negative body image experience negative affect, dysfunctional cognitions, and physiological arousal when confronted with their own body (Cash, 2011; Bauer et al., 2017). Body image as a construct can be subdivided into four components (Cash, 2004; Vocks et al., 2018). The first component includes the way the individual perceives her/his own body dimensions (i.e., perceptive component). Thoughts and emotions concerning the body, e.g., body dissatisfaction as the cognitive-affective manifestation of a negative body image (Thompson et al., 1999; Svaldi et al., 2012; Grogan, 2016) are summarized in the cognitive and affective components. The behavioral component manifests in body-related behaviors such as body avoidance behavior (i.e., avoidance of weighing, of seeing oneself in a mirror or of wearing tight clothes) and in BC (Vocks et al., 2006; Meyer et al., 2011; Legenbauer and Vocks, 2014).

Various researchers have found associations between BC and symptoms of clinically relevant eating pathology, e.g., body dissatisfaction (Kraus et al., 2015), overvaluation of shape and weight (Trottier et al., 2015; Calugi et al., 2017), bulimic behaviors (Kachani et al., 2013), and dietary restraint (Reas et al., 2006; Lavender et al., 2013). In line with this, a recent meta-analysis revealed large effect sizes for BC in all types of EDs, indicating higher rates of BC in EDs relative to healthy controls (Nikodijevic et al., 2018). Studies comparing bulimia nervosa (BN), anorexia nervosa (AN), and binge eating disorder (BED) found higher frequencies of BC in patients with BN than in patients with AN (Calugi et al., 2006; Kachani et al., 2013) and the lowest frequencies in patients with BED (Mountford et al., 2007). Nevertheless, BC is not limited to clinically relevant ED patients. In healthy samples, researchers found similar positive associations between BC, eating pathology, and body dissatisfaction (Reas et al., 2002; Haase et al., 2011; Latner et al., 2012; Stefano et al., 2016; Bailey and Waller, 2017) as well as negative affect (Reas et al., 2002; Latner et al., 2012; Stefano et al., 2016). However, BC engagement tends to be less frequent in subclinical women than in women with ED diagnoses (Reas et al., 2002). Recent studies in subclinical samples suggest that BC functions as a risk factor for the development of clinical EDs (e.g., Hildebrandt et al., 2010).

These empirical findings underscore the important role of BC in theoretical frameworks on the development and maintenance of body image disturbances and eating pathology. For example, in their cognitive-behavioral model of eating disorders, Williamson et al. (2004) proposed that BC leads to an overall reduction of negative emotions such as fear of fatness or anxiety. This reduction of negative affect is assumed to act as a negative reinforcement of BC. In addition, the short duration of typical BC sessions prevents habituation to the feared stimulus, thus maintaining BC (Walker and Murray, 2012). Contradicting the assumptions of the cognitive-behavioral model, Kraus et al. (2015) found increased negative affect immediately after a BC episode rather than decreased emotions. However, a recent study revealed theory-consistent time courses of arousal and emotional states during and after episodes of BC. Specifically, whereas negative emotions and arousal were heightened during a BC episode, 15 min after the episode of BC they had subsided, supporting cognitive-behavioral theories (Wilhelm et al., 2018).

Further questions concerning effects of BC on the development and maintenance of EDs were addressed by Shafran et al. (2007), who conducted the first experimental study on BC, implementing a low BC and a high BC condition. Participants in the low BC condition had to allocate their attention to all body parts and describe them in a neutral, non-judgmental way. During the high BC condition, participants focused on individual “problem areas” (i.e., negatively valenced body parts). The authors found increased body dissatisfaction, feelings of fatness and self-critical thinking among participants in the high BC condition. The effects were short-lived and subsided after 30 min. These findings suggest causal relationships between BC and body dissatisfaction. The effects of experimentally inducing BC of positively valenced body parts remain unclear.

While various studies employing different designs (i.e., experimental or descriptive) have demonstrated the clinical and non-clinical relevance of BC in females, less research has been conducted on BC in male populations. Male BC strategies significantly differ from those in females, as males tend to evaluate their chest muscles and overall muscle leanness (Alfano et al., 2011) whereas females examine their body girth in the mirror (for a systematic review, see Walker et al., 2009; Kachani et al., 2013; Nikodijevic et al., 2018). Several authors propose different internalized body ideals as one explanation for the variation of BC across genders (Hildebrandt et al., 2010). In terms of gender-specific body ideals, women rather strive for thinness while men strive for muscular and lean bodies with reduced body fat (Pope et al., 2000; Dakanalis et al., 2015). Due to these different body ideals, women want to lose body weight whereas men aim to gain weight as muscle mass (Penelo et al., 2012). The ideal male body, however, is almost impossible to achieve for most men without the abuse of anabolic-androgenic steroids (Kouri et al., 1995). An evolving field of research is focusing on the mental disorder muscle dysmorphia (MD), in which men develop a pathological preoccupation with muscularity, resulting in an extreme pressure to strive for a hyper-muscular body (Olivardia et al., 2000; Rohman, 2009). As a consequence, individuals with MD also engage in BC as a dysfunctional strategy to gain information on muscle size and density (Olivardia, 2001; Walker et al., 2012; Winter and Buhlmann, 2013). Moreover, BC has been shown to be associated with the abuse of anabolic-androgenic steroids (Hildebrandt et al., 2010), higher shape and weight concerns, body dissatisfaction, and depression in men (Walker et al., 2009).

As is the case with women, body dissatisfaction in men is not limited to clinical populations. Research suggests that body dissatisfaction is becoming more common among men (Leit et al., 2002) and is rising over time (Gray and Ginsberg, 2007), even though men are more satisfied with their bodies than women overall (Cooper and Fairburn, 1983; Else-Quest et al., 2012; Engeln et al., 2013; Grogan, 2016). A pilot study on male BC revealed that body dissatisfaction is associated with an increased frequency of BC and an engagement in dysfunctional behaviors (Walker et al., 2009). Following up on these findings, Walker et al. (2012) experimentally investigated the effects of BC on state body image and state muscle dissatisfaction in a male sample by randomly assigning participants to a high BC or low BC condition. Participants were either instructed to check negatively valenced body parts or describe their body in a non-judgmental way. The results indicated increased body dissatisfaction regardless of the condition. From these findings, it may be concluded that the focus of attention during BC might not matter in the development of body dissatisfaction. Contradicting these findings, however, Cordes et al. (2017) reported that negative affect is correlated with the amount of time men dwell on their negatively valenced body parts, suggesting a crucial role of attentional focus in emotional outcomes. Accordingly, the role of the attentional focus (i.e., on negatively vs. positively valenced body parts) during BC remains unclear and seems to lead to different emotional outcomes between the genders (Shafran et al., 2007; Walker et al., 2012). A recent correlation study provided first insights into the relation between BC and negative affect among subclinical men and women. Solomun-Krakus and Sabiston (2017) found that BC was associated with body-related negative affect, i.e., guilt and shame, in diverse age groups and both genders, with women experiencing significantly higher levels of guilt and shame as well as higher frequencies of BC than men. So far, experimental studies on gender differences in emotional responses to BC are lacking. In their recent meta-analysis, Walker et al. (2017) reviewed the existing literature and pointed out the need for studies including males or mixed gender data in order to draw causal conclusions regarding the influence of gender on associations between BC and eating pathology, negative affect, and body dissatisfaction.

Previous research has demonstrated that BC is not only a psychopathological symptom in females and males with EDs, but is also a widespread behavioral trait across men and women in general (Olivardia et al., 2000; Farrell et al., 2004; Hildebrandt et al., 2010; Haase et al., 2011; Meyer et al., 2011; Winter and Buhlmann, 2013; Walker et al., 2017). Furthermore, BC characteristics of males and females seem to differ significantly, by either evaluating thinness or muscularity (Shafran et al., 2004; Alfano et al., 2011; Dakanalis et al., 2015). Therefore, BC has been targeted as a transdiagnostic symptom in females and males with EDs (e.g., Olivardia et al., 2000; Kraus et al., 2015) and as a common phenomenon in non-clinical females and males (e.g., Shafran et al., 2007; Walker et al., 2012). However, no study to date has experimentally investigated gender differences in emotional and evaluative consequences of BC. Moreover, results of studies on BC of differently valenced body parts are conflicting (Shafran et al., 2007; Smeets et al., 2011; Walker et al., 2012; Cordes et al., 2015). A better understanding of post-BC negative affect and body satisfaction depending on valence could yield new approaches for interventions to improve body image. In order to fill the research gaps, the aim of the present study was to test gender differences in changes of negative affect and evaluated state body satisfaction induced by checking negatively and positively valenced body parts. For this purpose, non-clinical males and females underwent the checking of their positively valenced body parts, their negatively valenced body parts and a control condition in randomized order. Group differences in negative affect and state body satisfaction from pre- to post-BC, as well as the influence of the individual’s eating pathology, were investigated.

Hence, the present study had four aims: The first aim was to analyze emotional changes from pre- to post-BC of differently valenced body parts (i.e., subjectively positive vs. negative body parts). The second aim focused on changes in state body satisfaction from pre- to post-BC. Third, we investigated gender differences in negative affect and state body satisfaction from pre- to post-BC. Fourth, we analyzed eating pathology as a possible predictor of negative affect and state body satisfaction post-BC. Based on the literature outlined above, we expected that participants would show significantly greater changes in negative affect after focusing on negatively valenced body parts than on positively valenced body parts. Concerning the second aim, we hypothesized greater changes in state body satisfaction through performing BC of negatively valenced body parts compared to BC of positively valenced body parts. Third, we postulated that women would experience significantly higher negative affect and a less positive state body satisfaction compared to men. Finally, we assumed that the level of post-BC negative affect and post-BC state body satisfaction could be predicted by the individual’s eating pathology after controlling for pre-BC negative affect and pre-BC state body satisfaction.

## Materials and Methods

### Design

The present experimental study used a randomized crossover design with the within-subjects factors Condition (i.e., positive, negative and control) and Time (i.e., pre, post) and the between-subjects factor Gender (i.e., male, female) to address the stated hypotheses. The dependent variables measured were state body satisfaction and negative affect.

### Participants

Data were collected from *N* = 120 non-clinical participants (*n* = 60 women, *n* = 60 men). Pre-specified inclusion criteria were age between 18 and 50 years and body mass index (BMI) between 17.5 and 30 kg/m^2^. Persons who evaluated themselves as being suicidal and/or depressive or showed self-harm behavior were excluded. We further excluded participants who experienced binge eating episodes followed by compensatory vomiting more than once a week. Participants meeting other ED criteria, e.g., dietary behavior or frequent gym activities, were not excluded as these behaviors are common in healthy populations. Recruitment was carried out via public bulletin boards (e.g., in university buildings, fitness centers) and online announcements (i.e., students’ e-mail distribution list, social networks). After potential participants had been contacted via e-mail, they received a telephone call to determine study eligibility. Once they had passed the screening, participants were given information about the purported study content and the procedure. To disguise the purpose of the study, participants were told that it would investigate attentional processes regarding their own body. Thus, participants were unaware of the exact hypotheses of the study. As an incentive, participants either received study credit or 20 Euros. All participants provided written informed consent. The research project was developed in accordance with the Declaration of Helsinki and approved by the ethics board of Osnabrück University.

### Measures

#### Body Areas Ranking Scale (BARS)

To determine participants’ ranking of 14 body areas, they were asked to evaluate their own body areas in terms of relative satisfaction with each area using a questionnaire constructed for the purpose of the study. The 14 listed body areas were as follows: shoulders, breast, stomach, waist, hips, bottom, front of upper arms, back of upper arms, lower arms, upper back, lower back, front of thighs, back of thighs, and calves. The various body areas were presented in randomized order and had to be ranked hierarchically from 1 (most satisfied) to 14 (least satisfied) via a “drag and drop” system on a computer.

#### Body Image States Scale (BISS)

The BISS (Cash et al., 2002; German-language version Vocks et al., 2007a) was used to assess immediate changes in the cognitive-affective component of state body satisfaction. The scale encompasses a total of six items to evaluate current satisfaction with different aspects of physical appearance (e.g., body shape and weight). High scores indicate a positive state body satisfaction (i.e., high appearance satisfaction). Internal consistency was α = 0.91 for a female student sample (Vollstedt, 2013) and α = 0.62 for a male student sample (Cash et al., 2002). In the current study, Cronbach’s alpha ranged between α = 0.82 and α = 0.90 for females and between α = 0.78 and α = 0.82 for males.

#### Positive and Negative Affect Schedule Expanded Form (PANAS-X)

From the PANAS-X manual (Watson and Clark, 1994; German-language version Grühn et al., 2010), we chose the scale Guilt to assess negative affect pertaining to body image (e.g., guilt, shame, disgust), as previous research indicated that this scale is well suited to assess the emotional correlates of a disturbed body image (Solomun-Krakus and Sabiston, 2017). The scale comprises six items rated on a five-point Likert scale from 1 (not at all) to 5 (extremely). In the present study, for the three conditions and two points of measurement, internal consistencies ranged from α = 0.42 to α = 0.86.

#### Eating Disorder Examination-Questionnaire (EDE-Q)

The EDE-Q (Fairburn and Beglin, 2008; German-language version Hilbert and Tuschen-Caffier, 2006) was applied to assess eating pathology. The global EDE-Q score was calculated via mean scores of the four subscales Restraint, Shape Concern, Weight Concern, and Eating Concern and was used for the regression analyses. The scale consists of 22 items referring to typical symptoms of ED, which are rated on a seven-point Likert scale from 0 (no days/none of the times/not at all) to 6 (every day/every time/markedly). For the whole sample included in the present study, the internal consistency for the EDE-Q scores of the subscales amounted to α = 81 for Shape Concern, α = 0.45 for Eating Concern, α = 0.72 for Weight Concern, and α = 0.71 for Restraint. The internal consistency for the global EDE-Q amounted to α = 81.

#### Socioeconomic Data

Each participant provided information on the following body-related personal data: age, exercise (in hours per week), height, and weight. We calculated the BMI by dividing weight (kg) by height (m^2^). Additionally, the individual’s body fat was measured on a body-fat scale.

### Stimulus Material

#### Audio Files

To conduct BC tasks in a standardized manner, audio files with instructions for the 14 relevant body parts were recorded prior to data collection. Based on the individual ranking for the 14 body parts, two sets of standardized audio instructions were merged for each participant. Each individual set contained instructions for the four body parts with which the respective participant was most satisfied and the four body parts with which she or he was least satisfied. Instructions and wording for BC were based on items of the Body Checking Questionnaire (Reas et al., 2002), the Male Body Checking Questionnaire (Hildebrandt et al., 2010) and a manual for progressive muscle relaxation (Bernstein and Borkovec, 1995). Instructions were worded in a gender-neutral manner and included identical content and wording for each body part. The final instruction files lasted for about 15 min, including introduction and closure. For example, the audio instruction for the stomach was phrased as follows: *“Look at your stomach… Relax it completely… What shape does it have?…How does it look in the mirror?…Try to feel bones or muscles with your fingers… Pinch your stomach… Look in the mirror at the tissue that you have between your thumb and index finger… Now tense your stomach… In addition, pull your stomach in or arch it forward… Does this make the shape of your stomach change in the mirror?…Do muscles stand out under the skin?…Touch it to check how hard it is… Relax it again… Now try to pull the skin back so that muscles or bones stand out well underneath … Look at your stomach in the mirror while you’re doing this… Now take the tape measure that is hanging to your right on the mirror… Use it to measure the circumference of your stomach at its widest part… Then hang the tape measure back up… Finally, quickly bounce up and down onto your tiptoes several times… How does your stomach look in the mirror when you’re doing this?”*

#### Body-Checking Equipment

Body checking was carried out in front of a triptych mirror that was constructed for the purpose of the study. Due to its special construction, participants were able to examine themselves from a front, side, and back view, depending on the particular instruction, experiencing a 360° view of their own body. The three parts of the mirror were of identical size, with a height of 2.12 m and a width of 0.92 m. The two side wings were attached to the front mirror at an angle of 90°. The cross on which participants had to stand during the two mirror procedures was located at a distance of 0.55 m to the front mirror and 0.46 m to the side wings. Additionally, a tape measure was available to carry out the instructed measurements of body parts for BC purposes.

### Procedure

The data collection was divided into two parts, an online assessment and a laboratory assessment. The online assessment included a web-based collection of demographic and body-related data as well as questionnaires on eating pathology, body satisfaction and ranking of body parts. It took about 30 min to complete and was conducted at the participants’ home a few days prior to the laboratory assessment. The laboratory assessment consisted of three consecutive parts and took place in the laboratory of the Department of Clinical Psychology and Psychotherapy of Osnabrück University.

Within the laboratory assessment, participants underwent three conditions (“positive body checking,” PBC; “negative body checking,” NBC; “control body checking,” CBC) in a randomized order to avoid sequence effects. In the PBC condition, participants were asked to check subjectively positive body parts. In the NBC condition, the participants checked subjectively negative body parts. In the CBC condition, participants played the popular computer game “Frogger” for 15 min. To re-establish baseline levels of negative affect and body satisfaction and to avoid carry-over effects, the animated Disney movie “Planes” was split into three 30-min sequences, which participants watched before each condition. Both “Frogger” and “Planes” did not contain any depiction of human bodies. Before and after each condition, the state questionnaires BISS and PANAS were administered. During the NBC and PBC, participants were alone in the laboratory wearing a standardized set of gray underwear which consisted of a tube top and briefs for women (label: Lascana) and boxer shorts for men (label: H&M). Participants had to stand on the fixed cross in front of the three-part mirror. The audio instructions for BC were started by each participant on a computer. Participants were either instructed to examine the four body parts with which they were most satisfied in PBC or least satisfied in NBC. The order of assessed body parts, i.e., beginning with PBC or NBC, was determined at random (i.e., coin toss). Following the sample instructions, participants examined their bodies by pinching, flexing or measuring body parts. Instructions began with the highest-ranked body part and proceeded stepwise to the rank below. After completion, participants’ height, weight, and body fat were assessed on a body fat scale and they received their chosen compensation. The laboratory assessment lasted for a total of approximately 3 h. To protect privacy and ensure standardized instruction of BC and avoidance of experimenter effects, the experimenter was not present in the room during the laboratory assessment.

### Data Analysis

Data analysis was conducted using the software IBM SPSS Statistics Version 25. The significance level was set at α = 0.05, with Bonferroni adjustments to control for the family-wise error rate. To check for differences between men and women concerning potential confounding variables, i.e., age, level of education, BMI, trait questionnaires, eating pathology, and pre-scores on the BISS and PANAS, independent samples *t*-tests were conducted. Hedges’ *g* was used as an effect size measure for group differences. In conventional classifications, effect sizes for partial η^2^ ($\eta_{p}^{2}$) are defined as $\eta_{p}^{2}$ = 0.01 (small), $\eta_{p}^{2}$ = 0.09 (medium), and $\eta_{p}^{2}$ = 0.25 (large) (Lakens, 2013).

To analyze the effects of the three conditions on the cognitive-affective components of body image (i.e., BISS and PANAS scores), two 3 × 2 × 2 mixed-design analyses of variance (ANOVAs) were conducted with the within-subjects factor Condition (i.e., positive, negative, control) and Time (i.e., pre, post) and the between-subjects factor Gender (i.e., male, female). To follow up significant interaction effects, a series of Bonferroni-adjusted *post hoc t*-tests with pairwise comparisons were conducted. Finally, to assess the impact of trait-like eating pathology as a possible confounding variable (i.e., EDE-Q scores) on the dependent variables (i.e., BISS and PANAS scores) for males and females, stepwise multiple hierarchical regression analyses were conducted.

## Results

### Sample Characteristics

The descriptive sample characteristics and group differences are shown in Table 1. As displayed, the groups differed significantly with respect to age, exercise, BMI and body fat. Males were older, exercised more, had a higher BMI, and had less body fat compared to females. Furthermore, female participants showed a significantly higher eating pathology than did males.

**Table 1:** Descriptive statistics and group comparisons regarding sample characteristics.

	Females (*n* = 59)		Males (*n* = 60)		Test statistics		
Variables	*M*	*SD*	*M*	*SD*	*df*	*t*	*p*
Age (years)^a^	21.87	3.726	23.20	3.118	118	−2.126	0.036
BMI (kg/m^2^)^b^	21.59	2.322	23.08	2.273	116	−3.534	0.001
Body fat (%)^c^	24.65	5.205	18.36	5.290	112	6.399	<0.001
Sport exercise (hours/week)^d^	3.40	1.879	5.07	2.852	118	−3.780	<0.001
**Eating Disorder Examination-Questionnaire (EDE-Q)**							
Restraint	0.79	0.861	0.80	1.035	117	−0.058	0.954
Eating concern	0.39	0.388	0.17	0.270	117	3.529	0.001
Weight concern	0.99	0.944	0.60	0.656	117	2.613	0.010
Shape concern	1.64	1.092	1.06	0.721	117	3.423	0.001
EDE-Q global score	0.95	0.676	0.66	0.554	117	2.597	0.044

*BMI, body mass index; M, mean; SD, standard deviation; df, degrees of freedom. ^a^Females n = 60; ^b^Males n = 59; ^c^Females and Males n = 57; ^d^Females n = 60.*

Moreover, females displayed significantly higher pre-BC negative affect (i.e., PANAS-Guilt) compared to males, *t*(118) = 2.41, *p* = 0.018. With regard to pre-BC state body satisfaction, *t*-tests of BISS pre-values revealed significantly higher mean scores of state body satisfaction in males than in females *t*(118) = −2.84, *p* = 0.005. An overview on descriptive pre-BC and post-BC values of BISS and PANAS can be found in Table 2.

**Table 2:** Descriptive statistics concerning pre-BC and post-BC values of BISS and PANAS.

	Females (*n* = 60)		Males (*n* = 60)	
Variables	*M*	*SD*	*M*	*SD*
**NBC**				
BISS-pre	5.85	0.958	6.23	0.850
BISS-post	5.16	1.530	6.01	1.311
PANAS-pre	1.18	0.341	1.08	0.193
PANAS-post	1.60	0.756	1.35	0.528
**PBC**				
BISS-pre	5.69	1.167	6.34	0.941
BISS-post	5.37	1.494	6.39	1.177
PANAS-pre	1.13	0.194	1.07	0.157
PANAS-post	1.43	0.589	1.18	0.255

*BC, body checking; NBC, negative body checking; PBC, positive body checking; BISS, Body Image States Scale; PANAS-Guilt, Positive and Negative Affect Schedule, subscale Guilt; M, mean; SD, standard deviation.*

The frequencies of the female and male participants’ positive and negative ranking of their body parts can be found in Table 3. For each participant, the four most positively ranked and four most negatively ranked body parts out of the total ranking of 14 body areas were taken into account. In addition, to evaluate most-liked and least-liked body parts of females and males, percentages of occurrence of the four positively or negatively ranked body parts were calculated.

**Table 3:** Absolute and relative frequencies of positive and negative rankings of body parts.

	Valence of ranked body part for females				Valence of ranked body part for males			
Body part	Positive		Negative		Positive		Negative	
	*f*	*rf*	*f*	*rf*	*f*	*rf*	*f*	*rf*
Shoulders	35	14.58^1^	5	2.08	22	9.17^3,4^	11	4.58
Breast/chest	10	4.17	15	6.25	17	7.08	20	8.33
Stomach	14	5.83	30	12.50^3^	11	4.58	29	12.08^1^
Waist	20	8.33	18	7.50	10	4.17	25	10.42^2^
Hips	6	2.50	31	12.92^2^	12	5.00	23	9.58^3^
Bottom	19	7.92	16	6.67	13	5.42	21	8.75^4^
Front of upper arms	17	7.08	11	4.58	25	10.42^2^	13	5.42
Back of upper arms	10	4.17	24	10.00^4^	20	8.33	15	6.25
Lower arms	30	12.50^2^	6	2.50	26	10.83^1^	10	4.17
Upper back	24	10.00^3^	7	2.92	18	7.50	18	7.50
Lower back	20	8.33^4^	12	5.00	6	2.50	15	6.25
Front of thighs	10	4.17	21	8.75	22	9.17^3,4^	12	5.00
Back of thighs	7	2.92	37	15.42^1^	15	6.25	12	5.00
Calves	18	7.50	7	2.91	23	9.58	16	6.67
*N*	240	100.00%	240	100.00%	240	100.00%	240	100.00%

*f, absolute frequency of positively/negatively ranked body parts; rf, relative frequency of positively/negatively ranked body parts (rf = f/N), N = 240; ^1^the most positive/most negative body part; ^2^the second most positive/second most negative body part; ^3^the third most positive/third most negative body part; ^4^the fourth most positive/fourth most negative body part.*

### Group Comparisons of Effects of Engagement in BC on Negative Affect

The 3 × 2 × 2 ANOVA with PANAS-Guilt scores to measure negative affect reached significance with respect to the main effects of Condition, *F*(1.58,186.71) = 17.60, *p* < 0.001, $\eta_{p}^{2}$ = 0.13, and Time, *F*(1,118) = 49.17, *p* < 0.001, $\eta_{p}^{2}$ = 0.29, qualified by a significant two-way interaction of Condition × Time, *F*(1.85,218.50) = 19.91, *p* < 0.001, $\eta_{p}^{2}$ = 0.14 and further by a three-way interaction of Condition × Time × Gender, *F*(1.85,218.50) = 3.13, *p* < 0.049, $\eta_{p}^{2}$ = 0.03. Pairwise comparisons revealed that NBC and PBC led to significant increases in PANAS-Guilt scores from pre- to post-BC (PBC: *p* < 0.001, *g*_av_ = 0.21; NBC: *p* < 0.001, *g*_av_ = 0.34), whereas PANAS-Guilt in the control condition showed no significant changes (CBC: *p* = 0.361, *g*_av_ = 0.02). Concerning the three-way interaction, pairwise comparisons revealed the following pattern: Significant changes with increased negative affect from pre- to post-BC in NBC and PBC were observed for both females (PBC: *p* < 0.001, *g*_av_ = 0.30; NBC: *p* < 0.001, *g*_av_ = 0.42) and males (PBC: *p* = 0.043, *g*_av_ = 0.11; NBC: *p* = 0.001, *g*_av_ = 0.26), a difference which did not reach statistical significance in CBC for either gender (females: *p* = 0.931, *g*_av_ = 0.00; males: *p* = 0.169, *g*_av_ = 0.04).

### Group Comparisons of Effects of Engagement in BC on State Body Satisfaction

The 3 × 2 × 2 ANOVA with mean BISS scores as dependent variable revealed a significant main effect of Time, *F*(1,118) = 38.79, *p* < 0.001, $\eta_{p}^{2}$ = 0.25, qualified by two significant two-way interactions of Time × Gender, *F*(1,118) = 15.65, *p* < 0.001, $\eta_{p}^{2}$ = 0.12, as well as Time × Condition, *F*(1.86,219.98) = 7.70, *p* = 0.001, $\eta_{p}^{2}$ = 0.06. Subsequent Bonferroni-adjusted pairwise comparisons indicated that women showed a significant decrease in terms of their state body satisfaction from pre- to post-BC (*p* < 0.001, *g*_av_ = 0.38), while men did not (*p* = 0.111, *g*_av_ = 0.08). In addition, *post hoc* comparisons revealed that state body satisfaction in the NBC significantly differed to state body satisfaction after the PBC (*p* = 0.001, *g*_av_ = 0.30), and the CBC (*p* = 0.001, *g*_av_ = 0.32). Furthermore, a significant main effect emerged for Condition, *F*(1.93,227.13) = 4.58, *p* = 0.012, $\eta_{p}^{2}$ = 0.04, and for the interaction between Condition × Gender, *F*(1.93,227.13) = 3.88, *p* = 0.024, $\eta_{p}^{2}$ = 0.03. Moreover, pairwise comparisons revealed that over all three conditions, men and women differed significantly in terms of state body satisfaction (NBC: *p* = 0.005, *g*_av_ = 0.62; PBC: *p* < 0.001, *g*_av_ = 0.83; CBC: *p* = 0.006, *g*_av_ = 0.55). The three-way interaction between the factors Condition × Time × Gender failed to reach statistical significance, *F*(1.86,219.98) = 2.56, *p* = 0.084, $\eta_{p}^{2}$ = 0.021.

### Influence of Eating Pathology

Multiple hierarchical regression analyses for both genders were calculated in order to predict post-BC negative affect and state body satisfaction (i.e., PANAS and BISS scores) based on pre-BC values and eating pathology in NBC (see Table 4). For male and female participants, trait eating pathology was a significant predictor of post-BC negative affect after controlling for pre-BC negative affect (males: *F*[2,59] = 15.04, *p* < 0.001; females: *F*[2,58] = 20.66, *p* = 0.001). In terms of post-BC state body satisfaction, the overall regression model reached statistical significance for males but not for females, indicating that post-BC state body satisfaction of males was influenced by eating pathology (males: *F*[2,59] = 33.93, *p* = 0.001; females: *F*[2,58] = 64.64, *p* = 0.092).

**Table 4:** Multiple hierarchical regression models for the prediction of state body satisfaction BISS and negative affect PANAS-Guilt after checking negatively valenced body parts (post-BC).

	BISS (post-BC)						PANAS-Guilt (post-BC)					
	*B*	*SE B*	*β*	*p*	Δ*R*^2^		*B*	*SE B*	*β*	*p*	Δ*R*^2^	
**Females**												
*Step 1*					0.68	^∗∗^					0.30	^∗∗^
Constant	−1.29	0.59					0.18	0.30				
Pre-BISS	1.10	0.10	0.83	^∗∗^			1.21	0.25	0.55	^∗∗^		
*Step 2*					0.02	n.s.					0.13	^∗∗^
Constant	0.27	1.01					−0.33	0.31				
Pre-BISS	0.96	0.13	0.72	^∗∗^			0.94	0.24	0.43	^∗∗^		
EDE-Q	−0.38	0.22	−0.17	0.09			0.42	0.12	0.38	^∗∗^		
**Males**												
*Step 1*					0.45	^∗∗^					0.06	n.s.
Constant	0.33	0.83					0.61	0.38				
Pre-PANAS	0.91	0.13	0.67	^∗∗^			0.68	0.35	0.25			
*Step 2*					0.09	^∗∗^					0.28	^∗∗^
Constant	2.30	0.96					−0.07	0.35				
Pre-PANAS	0.79	0.13	0.59				0.52	0.30	0.19			
EDE-Q	−0.74	0.22	−0.31	^∗∗^			0.51	0.10	0.54	^∗∗^		

*BISS, Body Image States Scale; PANAS-Guilt, Positive and Negative Affect Schedule, subscale Guilt; EDE-Q, Eating Disorder Examination-Questionnaire, global score; B, regression weights; SE B, standard errors of the regression weights; β, standardized regression weights; ΔR^2^, percentage of variance explained; n.s., not significant; ^∗∗^p < 0.001.*

## Discussion

The present study investigated gender differences in emotional and evaluative changes in response to BC of positively and negatively valenced body parts. We hypothesized that the changes in negative affect would be greater from pre- to post-BC in NBC compared to PBC. Additionally, we expected that women would show greater changes in negative affect and state body satisfaction from pre- to post-BC compared to men. To our knowledge, our study was the first to use a crossover experimental design executing BC of differently valenced body parts and analyzing changes in state body satisfaction and negative affect in both men and women. Extending previous findings (e.g., Jansen et al., 2016; Solomun-Krakus and Sabiston, 2017; Walker et al., 2017), we found causal effects of BC on state body satisfaction and negative affect depending on valence, gender, and the extent of eating pathology.

The first aim of the study was to investigate changes in negative affect after the checking of differently valenced body parts. In support of previous findings and theoretical assumptions (Kraus et al., 2015; Solomun-Krakus and Sabiston, 2017), the checking of negatively valenced body parts led to a significant change in negative affect (i.e., guilt and shame). In line with our expectations, the checking of negatively valenced body parts resulted in heightened negative affect. These findings contradict the assumptions of the cognitive behavioral model of eating disorders, according to which BC should be followed by a reduction of negative emotions (Williamson et al., 2004). However, our results confirm a recent finding on the time course of negative affect resulting from BC, which showed that negative affect is initially increased, but decreases in the longer term (Wilhelm et al., 2018). Given that state alterations of negative affect were only examined once after each experimental condition, future research should consider employing several post-treatment measurements to analyze time courses of negative affect after BC, as differences concerning gender might develop over time.

Perhaps more surprisingly, the checking of positively valenced body parts also led to a significant increase in negative affect, thus contradicting our hypothesis, as we assumed no alteration of negative affect in the PBC. At first glance, this also seems to contradict a finding by Jansen et al. (2016), who reported positive effects of mirror exposure to participants’ self-defined attractive body parts. Nevertheless, this finding does confirm and extend the results of a study by Walker et al. (2012), who found an unexpected increase in negative affect in a non-judgmental condition in which male participants were asked to neutrally examine their bodies in the mirror. It thus seems that the checking of both positively valenced and negatively valenced body parts leads to increases in body-related negative emotions. Various possible explanations for this might be considered. First, in the present study, exposure only lasted for 15 min, whereas in the study by Jansen et al. (2016), participants were asked to describe their bodies in a self-enhancing, positive way for 30 min. Another possible explanation relates to the BC strategies utilized in the PBC task, e.g., mirror checking, measuring and pinching. These BC behaviors may all be considered as having the negative intention to find something unsatisfactory, even in the positively valenced body parts. By contrast, BC behaviors involving more positive intentions, e.g., gentle touch, caressing, relaxation methods, may result in different affective states and should be considered for future studies. Previous research suggests that negative emotional responses in the course of body exposure gradually decrease over a period of half an hour or more (Vocks et al., 2007b). Accordingly, PBC might have initially activated negative affect, which would have been reduced in the longer term. Moreover, Jansen et al. (2016) conducted five sessions in which a therapist was present and guided the positive verbalization. In the present sample, participants attended a single PBC session guided by audio instructions without any requirement to verbalize. Therefore, the emotional responses to the checking of positively valenced body parts appear to differ from the emotional responses to positive self-talk while looking at positively valenced body parts. It can be speculated that guided positive self-talk effectively controls self-critical thoughts which might arise immediately after BC (e.g., Shafran et al., 2007) and act as a cognitive mediator of negative affective responses. It would therefore be interesting to empirically explore body-related cognitions during the confrontation with one’s subjectively positive body parts via a think-aloud approach (e.g., Kollei and Martin, 2014). An implication of these findings for cognitive-behavioral body image therapy aiming to redirect attention toward subjectively positive body areas might be as follows: Therapists could integrate cognitive techniques to ensure that positive body-related thoughts are thoroughly practiced and verbalized. As a conclusion, the emotional response to a short body confrontation with both visual and haptic elements, conducted alone, seems to differ from the emotional response to a longer and guided confrontation with positive verbalizations, e.g., Jansen et al. (2016). Furthermore, to gain a full understanding of the emotional response to BC of positively valenced body parts, it is important to also measure positive affect in future studies. As the current study only examined changes in negative affect, i.e., guilt and shame, conceivable changes in positive affect remain unexplored.

Contrary to our hypothesis, the effects occurred in both subclinical females and males, i.e., we did not find a more pronounced affective reaction in females (e.g., Else-Quest et al., 2012). This further suggests that BC might impact the development and maintenance of body image disturbances and EDs not only in female populations (Stefano et al., 2016; Nikodijevic et al., 2018) but also in male populations (Walker et al., 2017). Contradicting the commonly held belief that women are generally more emotional than men (e.g., Brody and Hall, 2008; Barrett and Bliss-Moreau, 2009), men and women seem to display similar emotional responses to BC. However, environmental context and gender stereotypes play an important role in the occurrence of gender differences (Bussey and Bandura, 1999; Hyde, 2005): Various meta-analytic reviews have provided evidence that the magnitude and direction of gender differences depends strongly on the context (e.g., Eagly and Crowley, 1986; Anderson and Leaper, 1998; LaFrance et al., 2003). Our results provide first evidence that guilt and shame seem to be experienced by both males and females when confronted with full-body mirrors. As BC and related negative affect seems to play an important role in the context of body image disturbances in both genders, it is important to interpret these findings from a transdiagnostic perspective, also including body dysmorphic disorder (BDD), in which BC is a major symptom (Wilhelm et al., 2014). Compared, generally speaking, to concerns about one’s shape or weight in the case of EDs and MD, men and women with BDD most commonly report concerns about their skin, followed by hair and nose concerns (Phillips and Diaz, 1997). As a consequence, more than 90% of patients with BDD engage in compulsive behaviors such as mirror gazing of their perceived defects, i.e., BC (Veale and Riley, 2001). A recent study demonstrated the transdiagnostic mechanisms of BC, revealing that it led to a significant reduction of negative affect from pre- to post-BC, i.e., 15 min and 60 min after the checking episode, in BDD and ED patients (Hartmann et al., 2018a). In view of the transdiagnostic mechanisms of BC, performing BC of one’s own negatively valenced body parts might also lead to increased negative affect in BDD. Therefore, in order to experimentally investigate BC in the context of BDD in future studies, the individual checking strategies focusing on negatively valenced body parts should be experimentally induced, as these seem to differ from those commonly reported by patients with EDs. Furthermore, future studies on BC in clinical populations should screen patients for EDs, MD and BDD in order to analyze possible dissimilarities concerning BC strategies as well as potential maintaining factors of body image disturbances in these disorders.

Our second aim was to investigate state body satisfaction after the checking of differently valenced body parts. In line with current research (e.g., Jansen et al., 2016) and our hypothesis, NBC led to greater declines in state body satisfaction than did PBC. Consequently, the valence of checked body parts seems to play a decisive role in determining the effects of BC on state body satisfaction. The effects of PBC seem to differ in terms of negative affect and body satisfaction: While PBC did heighten negative body-related emotions, i.e., guilt and shame, no significant worsening of body satisfaction occurred. It can be suggested that the link between BC and negative affect might be more immediate than that between BC and body satisfaction. Although highly speculative, it is conceivable that shame constitutes an automatic and possibly biologically predisposed response (Sznycer et al., 2018) to the confrontation with one’s own body *per se*, irrespective of the specific valence of a checked body area. Changes in state body satisfaction might therefore not be as sensitive to BC as changes in negative body-related affect. Another possible explanation is that changes in body satisfaction might occur as a delayed response to BC and could therefore not be captured immediately after the checking. The results extend the findings of several authors (e.g., Walker et al., 2017; Nikodijevic et al., 2018), which suggested a causal link between BC and body dissatisfaction as clinically relevant symptoms of EDs. Furthermore, female and male individuals differed regarding BC-induced changes in state body satisfaction. Matching our predictions and adding to current knowledge on BC (e.g., Solomun-Krakus and Sabiston, 2017), BC led to a less positive state body satisfaction in women but not in men. As expected, these results highlight significant gender differences concerning the level and stability of body satisfaction. First, our results indicate that men are more satisfied with their bodies in general, as they display higher levels of body satisfaction than women in all conditions and at all time points (e.g., Pingitore et al., 1997). Second, men seem to possess a more stable body image than women, as no significant worsening of state body satisfaction occurred after confrontation in PBC and NBC. Therefore, the BC procedure does not seem to be as aversive for men as it is for women. In this regard, findings from the study highlight that men seem to possess higher body satisfaction than women overall – at both the state and trait level. These disparities between males and females might inform future research on gender-specific effects of ED treatment. Specifically, male patients with ED diagnoses might profit from therapeutic techniques to attenuate negative affective responses to one’s own body (e.g., exposure with a habituation rationale) or from improving emotion regulation rather than from targeting cognitive aspects of body image. Our finding complements previous findings from studies showing that emotion regulation difficulties contribute to disordered eating in non-clinical men (Lavender and Anderson, 2010; Griffiths et al., 2014).

The four most negatively rated body parts within the sample of female participants were, in hierarchical order, “back of thighs,” “hips,” “stomach,” and “back of upper arms.” In fact, these negatively valenced body parts are consistent with those in females with EDs, who reported “thighs,” “hips,” and “stomach” as their most disliked body parts (e.g., Jansen et al., 2005; Bauer et al., 2017). Male participants within our sample stated “stomach,” “waist,” “hips,” and “bottom” as their most negatively valenced body parts. To our knowledge, no study has yet investigated disliked body areas in an MD sample. In a supplementary analysis of data from a recent study by our research group, we found that males with MD also reported “stomach” as their most disliked body part, followed by “chest,” which was the fifth most negatively valenced body part in the healthy male sample (data from Waldorf et al., 2019). As a conclusion, “problem areas” might be identical between healthy females and females with an ED diagnosis, and between healthy males and males with an MD diagnosis.

Our third aim was to investigate the influence of eating pathology on BC-induced emotional and evaluative responses. Consistent with previous research, males reported less eating pathology than did females (e.g., Stanford and Lemberg, 2012). Integrating our results into previous research on gender differences in terms of behavioral symptoms of EDs (e.g., Striegel-Moore et al., 2009), negative affect post-BC was predicted by eating pathology in males and females: Males and females with higher scores in eating disorder symptoms tended to experience more negative affect from pre- to post-BC compared to those with lower scores. Our results are in line with findings by Vocks et al. (2007a), who showed that participants with a negative body image were more vulnerable to changes in state body satisfaction than participants without body image concerns. Taking the individual’s trait-like body image into account, the higher the body image concerns, the greater the changes in body satisfaction might be. The findings provide support for the cognitive-behavioral model proposed by Williamson et al. (2002, 2004), as individuals with high trait-like eating pathology may judge body-related cues in a more negative pattern compared to individuals with low trait-like eating pathology. Consequently, they may be vulnerable to even minor changes in state body image and negative affect after BC. In addition, men with higher scores on the EDE-Q tended to respond with a greater decline in state body satisfaction from pre- to post-BC. Surprisingly, no such relationship between eating pathology and state body satisfaction could be found for females. A possible explanation for this non-significant effect might lie in the high intercorrelation between pre-BC and post-BC state body satisfaction scores in females. As post-BC BISS scores could be predicted to a large extent by pre-BC BISS scores, EDE-Q eating pathology failed to reach statistical significance and was therefore not an incrementally valid predictor in the model. In further research, it would be interesting to examine the influence of the specific facets of eating pathology, i.e., Restraint, Eating Concern, Weight Concern, and Shape Concern, as potential predictors of post-BC body-related negative affect and body satisfaction.

Some limitations of the present study need to be mentioned. First, the generalizability of our findings is limited due to the sample of young, non-clinical male and female individuals. Due to the standardized audio-instructed BC in the study, it was not possible to capture BC strategies other than checking in front of a triptych mirror. To take into account the individual’s BC strategies in future research, it also appears to be worthwhile to integrate (sub-)cultural developments into future BC assessments. For instance, current research documents that the drive for muscularity is highly prevalent in a subpopulation of female weight-trainers (e.g., Hartmann et al., 2018b). In line with that, the perceived female body ideal has changed significantly over time, shifting to an ideal which incorporates increased muscularity into the thin body ideal (Bozsik et al., 2018). This development might have implications regarding typical BC strategies of females as well as the prevalence of MD, and should be considered in future research. In addition, although BC is a frequently occurring behavior in the general population, the transferability of the results to a clinical population is unknown. As the sample consisted of female and male students, no structured clinical interview to determine clinical diagnoses was conducted. Symptoms of mental disorders can be expected to some extent in healthy populations (Auerbach et al., 2018); therefore, the sample might have included participants who met the criteria for clinical diagnoses. Besides being a symptom of EDs, BC is also a major symptom in BDD. Studies examining the impact of BC on body satisfaction and affect should also take into account possible clinical diagnoses, e.g., by performing clinical interviews. Moreover, research suggests that females and males differ in terms of disordered eating behavior (e.g., Walker et al., 2017). As the common instruments to assess pathological eating behavior encompass items which can be thought of as “female-centric,” because they ask for body-related thoughts and behaviors more typical for women than for men, the level of eating pathology in males might be misjudged, and moderator effects might therefore be underestimated (Murray et al., 2017). Specifically, men tend to engage in muscularity-oriented rather than body-fat-oriented disordered eating (Murray et al., 2011, 2017). Further research should therefore include gender-specific instruments in order to assess specific state body image effects in males and females. A further limitation of the study pertains to the ranked body areas of satisfaction. Participants with higher trait body dissatisfaction may be more dissatisfied with particular body parts compared to participants with lower trait body dissatisfaction, even if the same body parts are selected in the ranking. Therefore, the rankings are not necessarily equivalent across participants. In line with this, a positively ranked body part does not necessarily represent an actual positive evaluation of that body part. It is conceivable that participants with a global negative evaluation of their bodies may be dissatisfied with all body parts. Therefore, this plausible scenario should be considered in future studies in non-clinical as well as clinical populations. Another limitation concerning the methodology was the lack of follow-up assessment of negative affect and state body satisfaction, as the existing literature suggests a decrease of negative emotions 15–30 min after the checking episode (Wilhelm et al., 2018). In order to realize post-BC assessments in the present study, the experiment would have had to last for a total of approximately 5 h. We therefore refrained from such assessments due to the potential negative impacts on participants’ physical ability to concentrate, motivation to participate, and adherence to BC instructions over such a lengthy time period. In favor of higher reliability and validity of measurements, we thus decided on the shorter procedure. Finally, the instruction to only focus on one body area at a time had no objective control, as gaze behavior was not monitored and no experimenter was present in the same room during the performance of BC. Hence, the experiment should be replicated with an experimenter being present in the same room in order to control for correct performance of BC tasks. However, an experimenter present in the room might bias task performance as participants might perform BC tasks extra carefully or conversely, distractedly due to the observer. Hence, further research should include eye-tracking as an objective and sufficient method of assessing the attentional focus and task performance of participants during BC as a manipulation check. Given that the nature of BC differs highly between males and females, with males aiming to maximize muscularity and females focusing on minimizing adiposity (Hildebrandt et al., 2010; Alfano et al., 2011), future research could also induce male- as well as female-appropriate forms of BC.

## Conclusion

In conclusion, the present study is the first to experimentally examine both males and females in terms of responses to BC, differentiating between BC of positively vs. negatively valenced body parts. It adds to the very limited knowledge on BC-induced affective and evaluative gender differences (e.g., Walker et al., 2017). Men and women did not significantly differ in their body-related negative affect, i.e., their affective states of guilt, shame and disgust, from pre- to post-BC. In terms of body satisfaction, men compared to women reported being more satisfied with their bodies, as reflected in a state body satisfaction that remained stable after BC. Therefore, women seem to be more vulnerable to body image-related influences than men. Moreover, the present findings provide insights into the transdiagnostic factor of BC in males and females, thus contributing to the understanding of maintaining processes of body image disturbances and symptomatology of EDs. Future studies should investigate men and women with EDs in order to identify BC-related similarities and differences between non-clinical and clinical samples. A better understanding of the transition from non-clinical to clinically relevant BC might be helpful for the development of early prevention programs.

## Ethics Statement

The research project was developed in accordance with the Declaration of Helsinki and approved by the ethics board of Osnabrück University. All participants provided written informed consent.

## Author Contributions

MW planned and conducted the study. JT analyzed the data and wrote the first draft of the manuscript. BR and SV contributed to the conception and design of the study. All authors contributed to the compilation of the manuscript and read and approved the submitted version.

## Conflict of Interest Statement

The authors declare that the research was conducted in the absence of any commercial or financial relationships that could be construed as a potential conflict of interest.
